# Effectiveness of splitter plate to control fluid forces on a circular obstacle in a transient flow: FEM computations

**DOI:** 10.1038/s41598-022-17947-w

**Published:** 2022-08-10

**Authors:** Qurrat ul Ain, Rashid Mahmood, Jan Awrejcewicz, Imran Siddique, Afraz Hussain Majeed, Witold Pawłowski

**Affiliations:** 1 Department of Mathematics, Air University, PAF Complex E-9, Islamabad, 44000 Pakistan; 2 Department of Automation, Biomechanics and Mechatronics, Lodz University of Technology, 1/15 Stefanowskiego St., 90-924 Lodz, Poland; 3 Department of Mathematics, University of Management and Technology, Lahore, 54770 Pakistan; 4 Institute of Machine Tools and Production Engineering, Lodz University of Technology, Lodz, Poland

**Keywords:** Engineering, Mechanical engineering

## Abstract

The reliability of the usage of a splitter plate (passive control device) downstream of the obstacle, in suppressing the fluid forces on a circular obstacle of diameter $D = 0.1\;\;{\text{m}}$ is studied in this paper. The first parameter of the current study is the attachment of a splitter plate of various lengths $(L_{i} )$ with the obstacle, whereas the gap separation $(G_{i} )$ between the splitter plate and the obstacle, is used as a second parameter. The control elements of the first and second parameters are varied from $0.1$ to $0.3$. For the attached splitter plates of lengths $0.2$ and $0.3$, the oscillatory behavior of transient flow at $Re = 100$ is successfully controlled. For the gap separation, $0.1$ and $0.2$ similar results are obtained. However, it is observed that a splitter plate of too short length and a plate located at the inappropriate gap from the obstacle, are worthless. A computational strategy based on the finite element method is utilized due to the complicated representative equations. For a clear physical depiction of the problem, velocity and pressure plots have been provided. Drag and lift coefficients the hydrodynamic benchmark values are also evaluated in a graphical representation surrounding the obstacle’s peripheral surface as well as the splitter plate. In a conclusion, a splitter plate can function to control fluid forces whether it is attached or detached, based on plate length and gap separation between obstacle and plate, respectively.

## Introduction

During the last decade or so the subject of flow control has received considerable attention and is an evolving field of Fluid Dynamics. The flow wake behind the bluff structure can induce unsteady forces, which have the power to ruin the structure's contact with the flow field. At the same time, a small change in the configuration can lead to large engineering benefits like drag reduction, lift enhancement, or mixing enhancement. Often CFD simulation aims to control the fluid forces acting on the obstacles.

Flow control is an intriguing area with numerous real-world applications, especially in aerodynamics. The uneven wake that appears behind an obstacle causes undesirable structural oscillations, which can lead to fatigue failure. Controlling the wake phenomena could directly benefit a wide range of engineering applications, including skyscrapers, naval risers, bridges, columns, and a few sections of airplanes. The downstream flow regime is mostly determined by the bluff object's configuration and the inflow velocity (or the Reynolds number, Re). In the downstream wake zone, isolated shear layers from both upper and lower regions of the bluff item lift above a critical Re, producing a Von-Karman vortex street or alternating vortices shedding. As a result of the bluff object's oscillating drag force and lift force caused by periodic vortex shedding, a considerable pressure reduction occurs. Both the devices, passive (without external energy input) and active (with some externally added energy input) are control strategies to adjust the unstable wake region and decrease the unstable pressures, induced on the bluff items that have been proposed in the literature^1–3^.

The splitter plate is considered as a very effective and useful passive device for flow control that has been widely investigated in the literature. Splitter plates^4–9^, fairings^10–12^, flow control that is suction-based^13,14^, control over moving boundary layer^15^, the slits that are in a parallel position to the inlet flow^16^, the streamlining of structural geometry^17,18^, the helical strakes^19,20^, and many other add-on passive devices^21,22^ are among the most common control measures. Triantafyllou et al.^23^ pointed the absolute instability that directs the vortex dynamics and global wake unsteadiness in the flow is principally modified by these control techniques. Choi et al.^1^ hypothesized that removing or decreasing the local absolute unsteadiness in the separated shear layers and wake centreline section could result in substantial control over Von-Karman vortex shedding and a decrease in unstable forces.

Splitter plates are one of the many passive flow control devices, researched widely in the literature. In literature, the impact of detached or attached rigid splitter plates is investigated experimentally and numerically. Hwang et al.^24^ evaluated the impact of a detachable plate on a circular obstacle and found that forces induced on the cylinder were significantly lessening. By altering the position of the splitter plate, Akilli et al.^25^ broaden the prior experimental research to evaluate the impact of a detached splitter plate on the abolishment of vortex shedding. Ozono^26^ investigated the impact of a detached splitter plate behind a cylinder in an asymmetric arrangement on the length and position of the plate. He noticed that as the distance between the cylinder’s central point and the forefront of the downstream splitting plate increased, the drag coefficient began to fall. Serson et al.^27^ investigated detachable splitting plates and discovered a considerable reliance on high Reynolds numbers. A significant parameter is a nondimensional gap from the base point of the cylinder to the splitter's forefront that determines the wake behavior, according to experiments on the detached splitter plate Rockwell and Unal^28^. It has been reported by Cete and Unal^29^ that the drag coefficient of the attached type rigid splitter plate reduces monotonically as the length of the plate increases up to twice the cylinder's diameter. The length of the stiff splitter for which the vortex shedding phenomena is obstructed for flow through a circular cylinder is the subject of another investigation by Kwon and Choi^5^.

All the work discussed above is remarkable for the control of wake flow behind a cylinder by a splitter plate in a viscous flow. Among these various cylinder forms, the circular-shaped obstruction serves as a vital element of design and structure. These structures frequently contact with fluids and are subjected to forces induced due to flow, which might result in a disaster within some definite conditions. Recently, an experimental study by Chen et al.^30^ has been performed for a wind tunnel. They investigated the impact of various gaps between I adjacent passive jet rings for controlling the VIV. To investigate the plate length’s sensitivity Gao et al.^31^ provided an upstream splitter plate to the stagnation point of a circular cylinder. They concluded that the reduction in the mean drag and fluctuating lift could reach 36.0% and 63.6%, respectively, when L/D was equal to 1.0. To envisage the VIV response of a cylinder of circular shape immersed in a fluid, Chen et al.^32^ established, a coupled wake oscillator dynamic equation.

For our present study we considered, a landmark paper published by Schaefer et al.^33^ on obstacle flow, in which they investigated a benchmark problem for incompressible flow around a circular cylinder and built a comparison between results obtained using various solutions methodologies. The fundamental aim of the current work is to study the effects of a control splitter plate attached to the cylinder of circular shape on the reduction of drag and enhancement of lift at $Re = 100$, for a variety of attached plates of non-dimensional lengths $\left( {L_{i} , i = 0.1,\;0.2 \;{\text{and}}\; 0.3} \right)$ and also for detached splitter plate of fixed length $L_{3} = 0.3$ located at different positions $\left( {G_{i} , i = 0.1,0.2\;{\text{ and}}\; 0.3} \right)$. Moreover, all results for the present work are attained by FEM-based simulations. Mahmood et al.^34,35^ have done FEM computation-based study for analysis of the viscous fluid flow characteristics inside a channel idriven-cavity. Also, described the effects of shape function for linear as well as quadratic profiles. For the physical setup of the problem, different hybrid computational meshes based on FEM are described. The properties of fluid forces across the obstruction were also discussed.

The current work is separated into several categories and organised as follows: in the first and second sections, the problem is introduced, and the flow configuration and governing equations with constitutive relations are explained. The third section consists of the description of the numerical scheme and grid convergence. Moreover, for code validation, the results are compared with the literature. Whereas, all the results and discussion of the article are provided in the fourth section. The conclusions of this research work are presented in the last section.

## Physical problem and mathematical modeling

**Figure 1:**
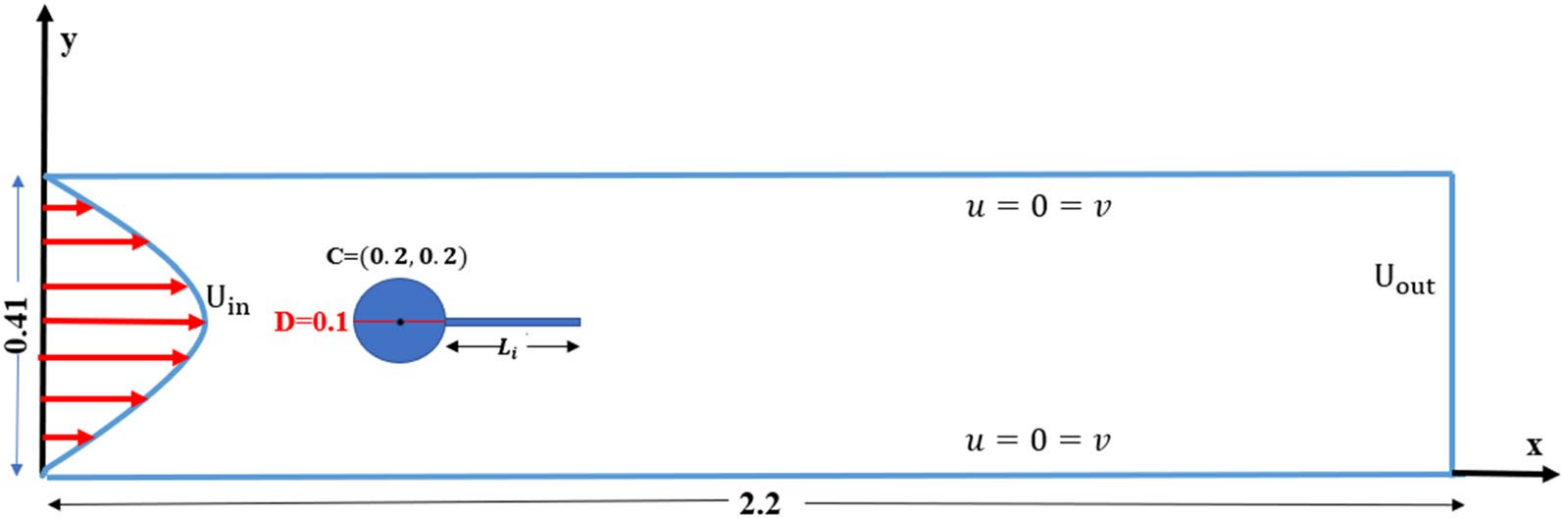
Schematic diagram, when a splitter of length $L_{i} ,where\; i = 1,2\;{ }and \;3$ is attached with the cylinder.

**Figure 2:**
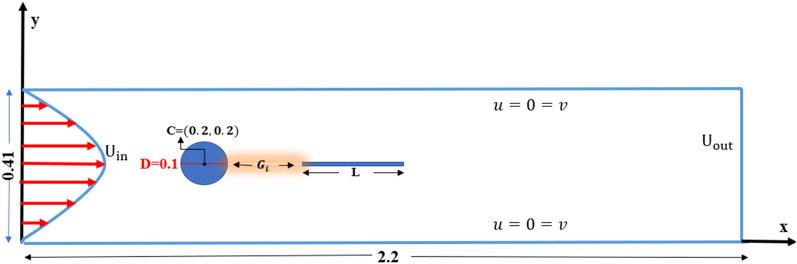
Schematic diagram, when a splitter of length $\tilde{L}$ is fitted at various gap separation $G_{i} , (where\, i = 1,2 \;and \;3$) from the cylinder.

Consider a flow which is transient, incompressible, two-dimensional viscous fluid have an interaction with circular cylinder located inside a channel. The height of the channel is $H = 0.41\;{\text{m}}$ and $L = 2.2\;{\text{m}}$ length is the dimension of the physical domain with a diameter of cylinder $D = 0.1\;{\text{m}}$ placed at $C\left( {0.2,0.2} \right)\;{\text{m}}$ are considered. The problem of laminar, incompressible, and unsteady flow past a circular cylinder is examined in this paper for two different cases: the cylinder with attached and the detached splitter plate. In the case of a splitter plate attached to the surface of the cylinder, plates of different lengths $L_{1} = 0.1, L_{2} = 0.2\; {\text{and}} \;L_{3} = 0.3$ are simultaneously introduced in the physical configuration as shown in Fig. 1. For the case of the detached splitter plate shown in Fig. 2, a spitter plate of fixed length $\tilde{L} = 0.3$ is located at three different positions, such as at gap $G_{1} = 0.1, G_{2} = 0.2, \;{\text{and}} \;G_{3}$, respectively. For all the cases, the plate thickness is the same, equal to 0.01 m.

For nondimensionalized governing equations, the average velocity $U_{ref}$ and length (cylinder diameter $D$) scales are employed, respectively.

For analysis, a few assumptions are made.

- The characteristics of transient flow for Newtonian fluid are studied.
- It is carried out, the impact of parabolic inlet flow while ignoring the impacts of body forces.
- Assuming the boundary condition (No-slip) on the cylinder’s surface and splitter plates as well as on both symmetric channel walls.

For a viscous, incompressible fluid in a transient flow across the circular obstacle along with a splitter plate i.e. attached or detached, the governing equations are the usual continuity and momentum equations. The dimensionless form of governing equations is defined below:

1 $$\overline{u}_{{\overline{x}}} + \overline{v}_{{\overline{y}}} = 0$$

2 $$\left[ {\overline{u}_{{\overline{t}}} + \left( {\overline{u}\overline{u}_{{\overline{x}}} + \overline{v}\overline{u}_{{\overline{y}}} } \right)} \right] = - \overline{p}_{{\overline{x}}} + \frac{1}{Re}\left[ {\overline{u}_{{\overline{x}\overline{x}}} + \overline{u}_{{\overline{y}\overline{y}}} } \right]$$

3 $$\left[ {\overline{v}_{{\overline{t}}} + \left( {\overline{u}\overline{v}_{{\overline{x}}} + \overline{v}\overline{v}_{{\overline{y}}} } \right)} \right] = - \overline{p}_{{\overline{y}}} + \frac{1}{Re}\left[ {\overline{v}_{{\overline{x}\overline{x}}} + \overline{v}_{{\overline{y}\overline{y}}} } \right]$$

The conditions imposed on boundary for transient flow field inside a channel with a circular obstruction, are described as:

Inlet boundary $$\overline{u}\left( {0,\overline{y},\overline{t}} \right) = \frac{{4U_{max} \overline{y}\left( {H - \overline{y}} \right)}}{{H^{2} }},\overline{v} = 0,$$

Outlet boundary $$\overline{u}_{{\overline{x}}} = \overline{v}_{{\overline{y}}} = 0,$$

Side walls $$\overline{u} = \overline{v} = 0,$$

Cylindrical surface $$\overline{u} = \overline{v} = 0$$.

The above non-dimensional iequations have been produced by adopting the scaling variables $L_{ref} ,U_{ref} ,$ the reference length and the average velocity, respectively.The dimensionless parameter Reynolds number $Re$ is defined by $Re = \frac{{U_{ref} L_{ref} }}{v}$ with $U_{ref} = \frac{2}{3}$ $U_{max}$ is the average velocity, $L_{ref} = D = 0.1$, $U_{max} = 1.5,$ $\nu = 0.001$ is kinematic viscosity^30^.

At the post-processing stage, we have deduced the quantities of interest described as:

- Drag coefficient
  4 $$C_{{\text{D}}} = \frac{{2F_{{\text{D}}} }}{{\rho U_{ref}^{2} D}},$$
- Lift coefficient
  5 $$C_{{\text{L}}} = \frac{{2F_{{\text{L}}} }}{{\rho U_{ref}^{2} D}},$$

The non-dimensional drag $\left( {F_{{\text{D}}} } \right)$ and lift $\left( {F_{{\text{L}}} } \right)$ forces act on the circular obstacle.

## Numerical procedure

FEM computation is used to achieve and handle a mathematical formulation of governing Eqs. (1)–(3). The stable finite element pair ${\mathbb{P}}_{2} - {\mathbb{P}}_{1}$ satisfying the inf–sup condition has been utilized. The Newton's iterative process is used to linearize the discrete non-linear systems^34,35^. For the solution of linearized system, PARDISO solver^36^ is utilized which works for general system $Ax = b$ and is based on $LU$ matrix factorization with special reordering of unknowns and reduce the number of iterations required to achieve the desired level of convergence. Furthermore, to stabilize the flow at higher values of $Re$, a cross-wind stabilization technique is implemented.

### Weak formulation

The basic mechanism for solving the system of Eqs. (1–3) is the ifinite element method. The initial step is conversion of the Eqs. (1–3) into what is known as “weak formulations.” We begin by introducing test and trial spaces, as follows:

Let $W = \left[ {H^{1} \left( \Omega \right)} \right]^{2}$ be the test subspaces for $\overline{u},\overline{v}$, and for pressure $Q = L^{2} \left( \Omega \right)$ is the test space. The weak form of the above equations is as follows:

6 $$\mathop \int \limits_{\Omega } \left( {\overline{u}_{{\overline{x}}} + \overline{v}_{{\overline{y}}} } \right)\,q\,d\Omega = 0,$$

7 $$Re\mathop \int \limits_{\Omega } \left( {\overline{u}_{{\overline{t}}} + \overline{u}\overline{u}_{{\overline{x}}} + \overline{v}\overline{u}_{{\overline{y}}} } \right)\,w\,d\Omega + Re\mathop \int \limits_{\Omega } \overline{p}_{{\overline{x}}} \,w\,d\Omega - \mathop \int \limits_{\Omega } \left( {\overline{u}_{{\overline{x}\overline{x}}} + u_{{\overline{y}\overline{y}}} } \right)\,w\,d\Omega = 0,$$

8 $$Re\mathop \int \limits_{\Omega } \left( {\overline{v}_{{\overline{t}}} + \overline{u}\overline{v}_{{\overline{x}}} + \overline{v}\overline{v}_{{\overline{y}}} } \right)\,w\,d\Omega + Re\mathop \int \limits_{\Omega } \overline{p}_{y} \,w\,d\Omega - \mathop \int \limits_{\Omega } \left( {\overline{v}_{{\overline{x}\overline{x}}} + v_{{\overline{y}\overline{y}}} } \right)\,w\,d\Omega = 0,$$

In above Eqs. (6)–(8) $q$ and $w$ are defined as test functions for pressure and velocity respectively.

In finite-dimensional subspaces, we compute continuous solutions with discrete ones for numerical approximation.

9 $$\begin{gathered} \overline{u} \approx \overline{u}_{h} \in W_{\hbar } \hfill \\ \overline{v} \approx \overline{v}_{h} \in W_{\hbar } \hfill \\ \overline{p} \approx \overline{p}_{h} \in Q_{\hbar } \hfill \\ \end{gathered}$$

Using (9) in (6)–(8), the following discrete version is obtained

10 $$\mathop \int \limits_{\Omega } \left( {\overline{u}_{{h_{{\overline{x}}} }} + \overline{v}_{{h_{{\overline{y}}} }} } \right)q_{\hbar } \,\,d\Omega = 0,$$

11 $$Re\mathop \int \limits_{\Omega } \left( {\overline{u}_{{h_{{\overline{t}}} }} + \overline{u}_{h} \overline{u}_{{h_{{\overline{x}}} }} + \overline{v}_{h} \overline{u}_{{h_{{\overline{y}}} }} } \right)\,w_{\hbar } d\Omega + Re\mathop \int \limits_{\Omega } \overline{p}_{{h_{{\overline{x}}} }} \,w_{\hbar } d\Omega - \mathop \int \limits_{\Omega } \left( {\overline{u}_{{h_{{\overline{x}\overline{x}}} }} + \overline{u}_{{h_{{\overline{y}\overline{y}}} }} } \right)w_{\hbar } \,\,d\Omega = 0,$$

12 $$Re\mathop \int \limits_{\Omega } \left( {\overline{v}_{{h_{{\overline{t}}} }} + \overline{u}_{h} \overline{v}_{{h_{{\overline{x}}} }} + \overline{v}_{h} \overline{v}_{{h_{{\overline{y}}} }} } \right)\,w_{\hbar } d\Omega + Re\mathop \int \limits_{\Omega } \overline{p}_{{h_{{\overline{y}}} }} \,w_{\hbar } d\Omega - \mathop \int \limits_{\Omega } \left( {\overline{v}_{{h_{{\overline{x}\overline{x}}} }} + \overline{v}_{{h_{{\overline{y}\overline{y}}} }} } \right)w_{\hbar } \,\,d\Omega = 0,$$

Basis function are defined for discrete solution as follows:

13 $$\begin{gathered} \overline{u}_{h} \approx \mathop \sum \limits_{k = 1}^{d.o.f} \overline{u}_{k} \varphi_{k} \left( {x,y} \right) \hfill \\ \overline{v}_{h} \approx \mathop \sum \limits_{k = 1}^{d.o.f} \overline{v}_{k} \varphi_{k} \left( {x,y} \right) \hfill \\ \overline{p}_{h} \approx \mathop \sum \limits_{k = 1}^{d.o.f} \overline{p}_{k} \psi_{k} \left( {x,y} \right) \hfill \\ \end{gathered}$$

where $d.o.f$ depicts the degrees of freedom.

Using Eqs. (10)–(12) give rise to

14 $$Re\mathop \int \limits_{\Omega } \left( {\overline{1}_{{h_{{\overline{t}}} }} + \overline{u}_{h} u_{{h_{{\overline{x}}} }} + \overline{v}_{h} \overline{u}_{{h_{{\overline{y}}} }} } \right)\,w_{\hbar } d\Omega + Re\mathop \int \limits_{\Omega } \overline{p}_{{h_{{\overline{x}}} }} \,w_{\hbar } d\Omega - \mathop \int \limits_{\Omega } \left( {\overline{u}_{{h_{{\overline{x}}} }} w_{{\hbar_{{\overline{x}}} }} + \overline{u}_{{h_{{\overline{y}}} }} w_{{\hbar_{{\overline{x}}} }} } \right)\,\,d\Omega = 0,$$

15 $$Re\mathop \int \limits_{\Omega } \left( {\overline{v}_{{h_{{\overline{t}}} }} + \overline{u}_{h} \overline{v}_{{h_{{\overline{x}}} }} + \overline{v}_{h} \overline{v}_{{h_{{\overline{y}}} }} } \right)\,w_{\hbar } d\Omega + Re\mathop \int \limits_{\Omega } \overline{p}_{{h_{{\overline{y}}} }} \,w_{\hbar } d\Omega - \mathop \int \limits_{\Omega } \left( {\overline{v}_{{h_{{\overline{x}}} }} w_{{\hbar_{{\overline{x}}} }} + \overline{v}_{{h_{{\overline{y}}} }} w_{{\hbar_{{\overline{x}}} }} } \right)\,\,d\Omega = 0,$$

16 $$\mathop \int \limits_{\Omega } \left( {\overline{u}_{{h_{{\overline{x}}} }} + \overline{v}_{{h_{{\overline{y}}} }} } \right)\,q_{\hbar } \,d\Omega = 0,$$

In the matrix form,

17 $$\left[ {\begin{array}{*{20}c} {M_{h} + Re.L_{h} + N_{h} \left( {\overline{u}_{h} ,\overline{v}_{h} } \right)} & 0 & {Re.B_{1} } \\ 0 & {Re.L_{h} + N\left( {\overline{u}_{h} ,\overline{v}_{h} } \right)} & {Re.B_{2} } \\ {B_{1}^{T} } & {B_{2}^{T} } & 0 \\ \end{array} } \right]\left[ {\begin{array}{*{20}c} {\overline{u}_{h} } \\ {\overline{v}_{h} } \\ {\overline{p}_{h} } \\ \end{array} } \right] = \left[ {\begin{array}{*{20}c} {F_{{\overline{u}_{h} }} } \\ {F_{{\overline{v}_{h} }} } \\ 0 \\ \end{array} } \right]$$

Here $M_{h}$ is the discrete mass matrix, $L_{h}$ is discrete Laplacian operator for the diffusion term and $N_{h}$ is the convective matrix at discrete level.

### Validation of results

**Figure 3:**
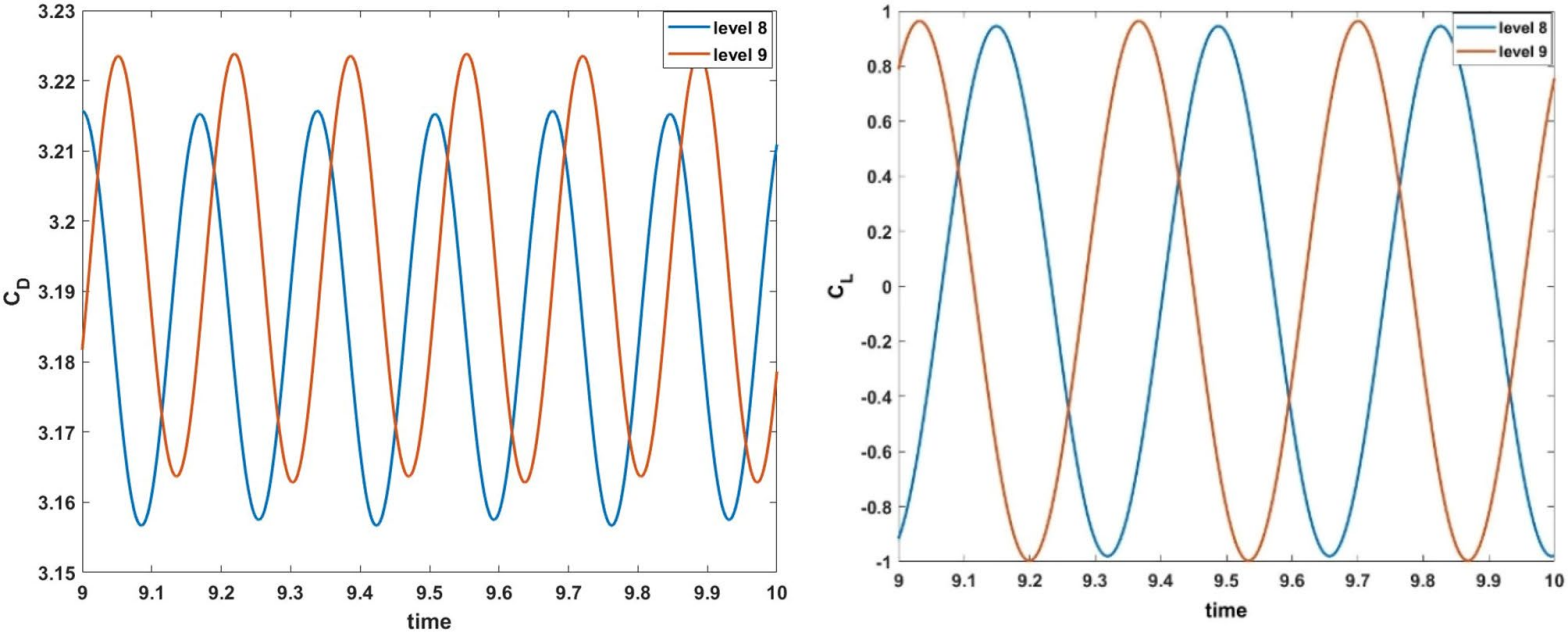
Drag and lift comparison tests for periodic flow at $Re = 100$,^33^.

The legitimacy of the numerical results must be established because the current numerical study is performed using a CFD programme based on PARDISO solver. For authentication of the present numerical investigation the drag coefficient and lift coefficient of a circular cylinder were evaluated to compare with previous studies and graphically shown in Fig. 3. From graphs it is observed that, amplitude increases with increment in refinement level. The latest results are very similar to what has already been reported numerically by Schafer et al.^33^, represented in Table 1.

**Table 1:** Comparisons between the present results and the available in the literature for the problem with a bare cylinder.

Research	Maximum of $C_{{\text{D}}}$	Maximum of $C_{L}$
Present work	3.2157	0.9635
Schäfer and Turek^33^	3.2224	0.9672

### Grid independence test

**Figure 4:**
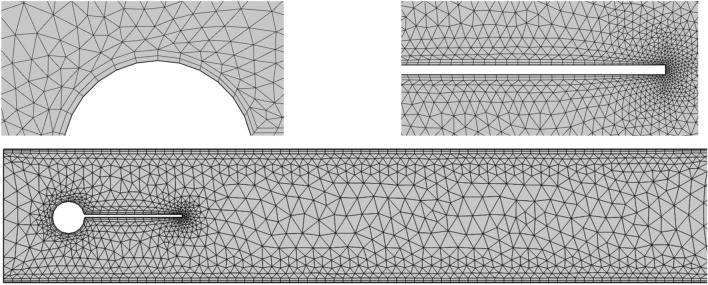
Hybrid mesh grid detail.

Mesh refinement is an important step for validation of a finite element model and gaining reader confidence in the work's physical results. Figure 4 demonstrates the computational domain that is meshed using the elements of triangular and quadrilateral shapes. Because of the velocity gradient high value, the quadrilateral elements iwere utilized near the walls of the cylinder and the edges of the splitter plate. Moreover, triangular elements were used for the remaining computational domain. The grid independence was tested on the flow across the circular shaped cylinder with an attached plate of length $L_{2} = 0.2.$ The mesh resolutions were varied to four different levels to observe the spatial convergence. By comparison of the numerical values in Table 2, it was observed for both case 1 and case 2 that the mesh resolutions were suitable to precisely estimate impact of splitter plater on the control of hydrodynamic forces. The refinement of grid resolution from case 2 to case 3, predicted more accuracy of results. However, as the mesh density is increased more as from case 3 to case 4 there was no considerable change on the numerical quantities observed.

**Table 2:** Test for grid independence using an attached splitter plate to the circular cylinder.

Cases	Refinement levels	Number of elements	Maximum of $C_{D}$
1	Normal	7366	2.779997
2	Fine	12,850	2.766796
3	Finer	29,706	2.766353
4	Extra fine	71,436	2.766178

## Results and discussion

A numerical study was conducted in a two-dimension channel to analyze the reduction in flow induced forces on a circular cylinder in existence of control passive device, placed at various downstream locations of the cylinder. Computations are carried out by applying FEM at a fixed Reynolds number of 100. This section presents the effects of control plates on flow around the cylinder are analyzed for two different arrangements, such that for the attached splitter plate of different lengths $L_{1} = 0.1$ to $L_{2} = 0.2$ and $L_{3} = 0.3$, and for detached plate at various gap separations $G_{1} = 0.1$, $G_{2} = 0.2$, and $G_{3} = 0.3$. The results are presented graphically for each case to show the effect of splitter plate for the reduction in drag force and to control the periodicity of lift force.

**Figure 5:**
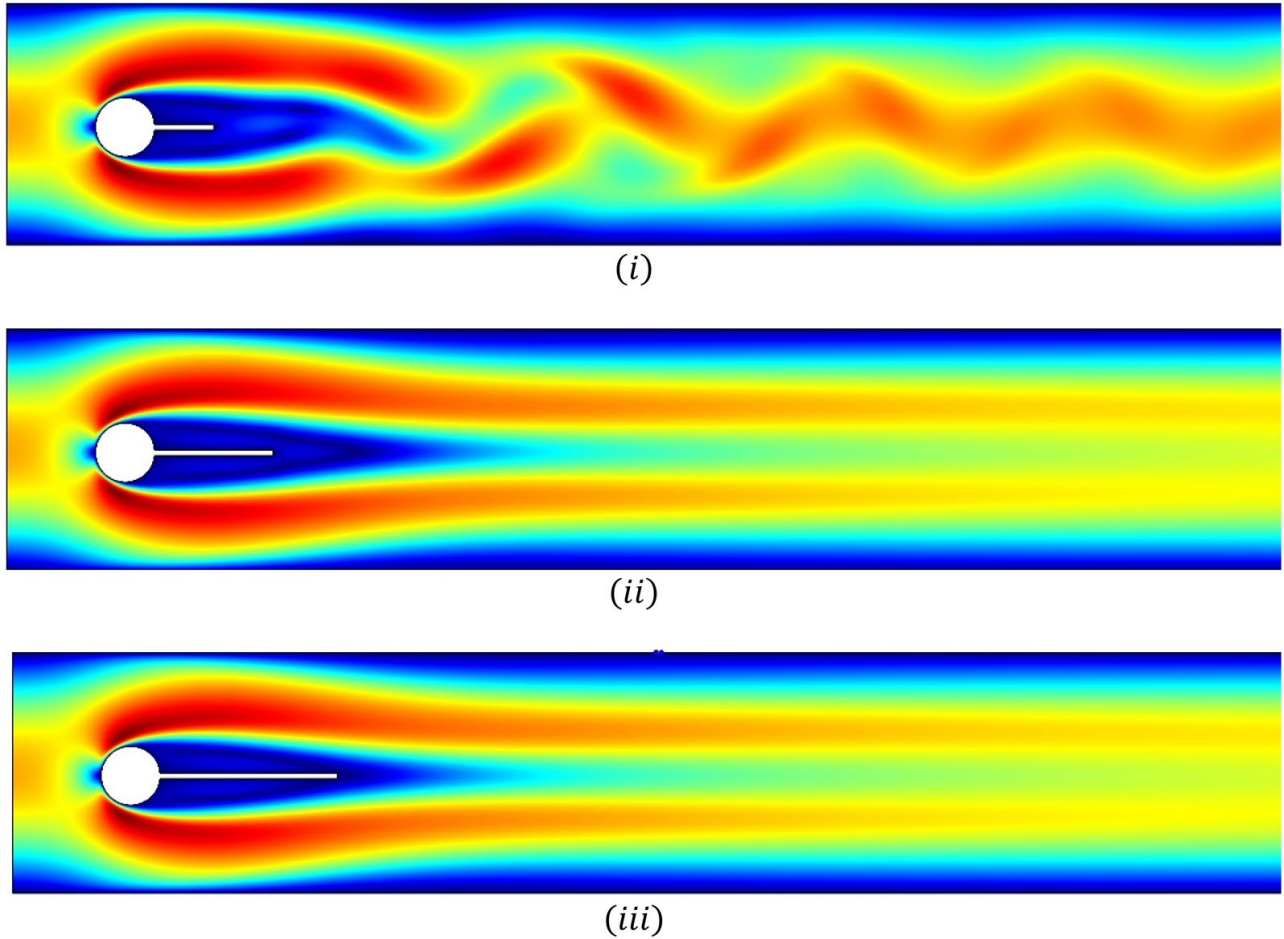
Influence of attached splitter plates on velocity, of lengths (**i**) $L_{1} = 0.1,$ (**ii**) $L_{2} = 0.2,$ (**iii**) $L_{3} = 0.3$ with $Re = 100$ at $\overline{t} = 10.$

**Figure 6:**
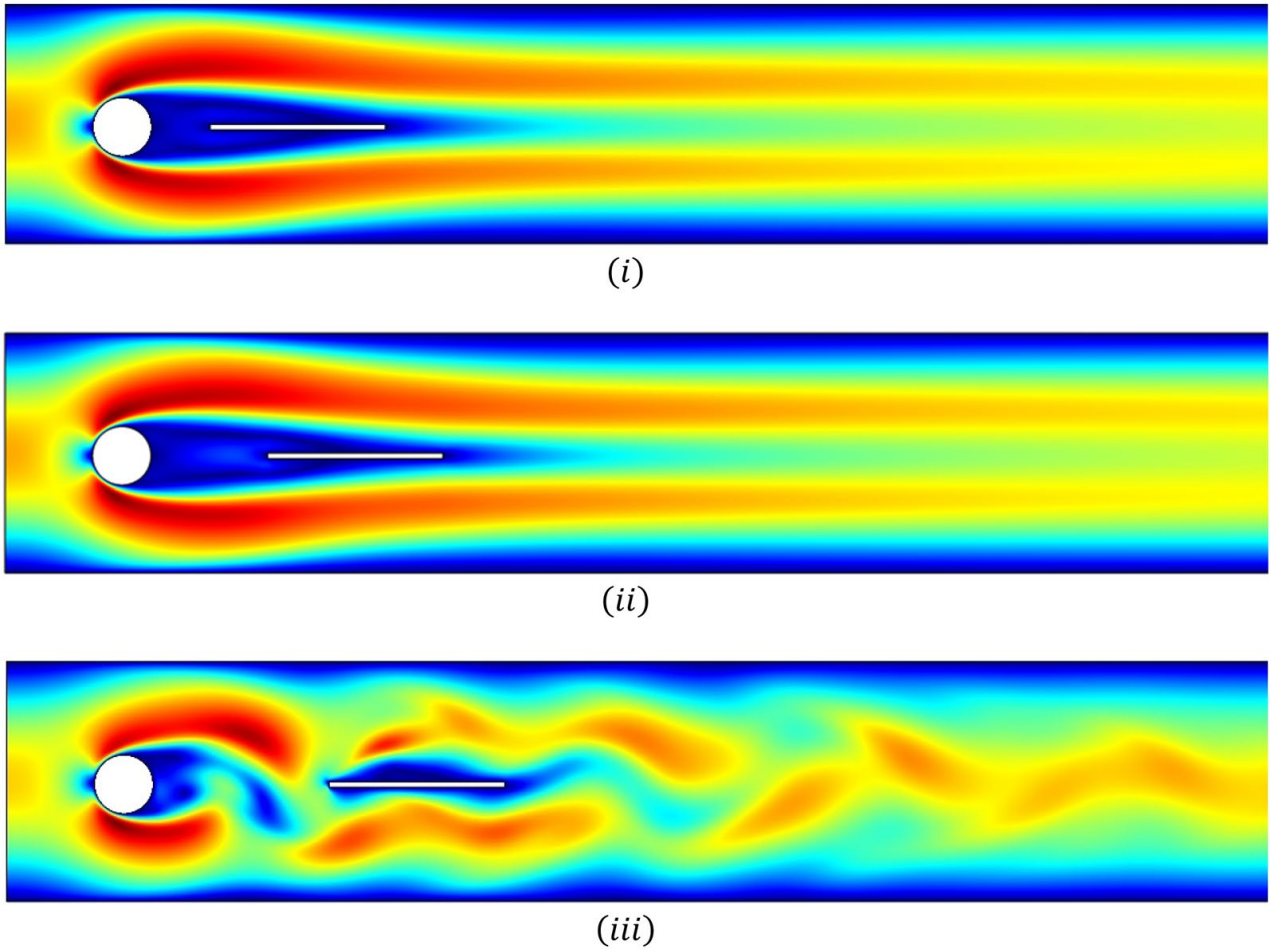
Influence of detached splitter plate (length $$L = 0.3)$$ on velocity, placed at gaps (**i**) $$G_{1} = 0.1,$$ (**ii**) $$G_{2} = 0.2,$$ (**iii**)$$G_{3} = 0.3$$ with $$Re = 100$$ at $$\overline{t} = 10.$$

Figures 5i–iii and 6i–iii are velocity profiles for the case when the splitter plate is attached to the circular cylinder and for detachment, respectively. The fluid begins with a parabolic velocity profile as an inlet velocity, and at a right angle the stagnation point region is created to the flow direction, and the fluid bifurcates about the circular obstacle with greater velocity, whereas the fluid so close to the side walls admits minimum velocity or zero velocity due to the no-slip condition. For the case $\left( I \right)$, it can be noticed from Fig. 5i that the behavior of fluid flow is periodic but by increasing the length of the attached splitter plate from $L_{1} = 0.1$ to $L_{2} = 0.2$ and $L_{3} = 0.3$ the periodicity is overcome. In case $\left( {II} \right)$, splitter plate of length $\tilde{L} = 0.3$, Fig. 6i, ii depict that by locating the plate at gap $G_{1} = 0.1$ and $G_{2} = 0.2$ periodicity is under control, while at gap $G_{3} = 0.3$ the splatter plate is unable to overcome periodic flow, shown in Fig. 6iii.

**Figure 7:**
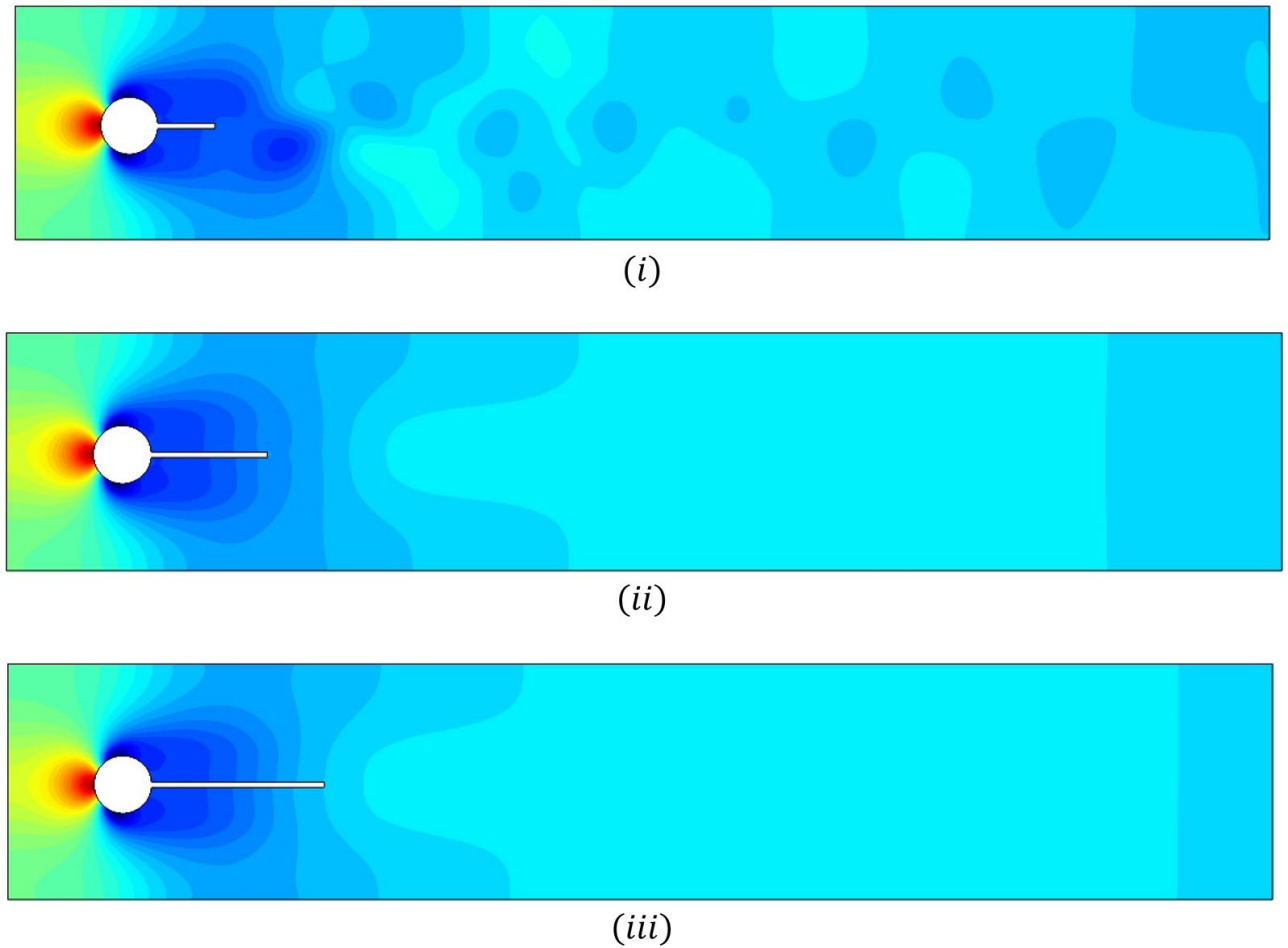
Influence of attached splitter plates on pressure, of lengths (**i**) $L_{1} = 0.1,$ (**ii**) $L_{2} = 0.2,$ (**iii**) $L_{3} = 0.3$ with $Re = 100$ at $\overline{t} = 10.$

**Figure 8:**
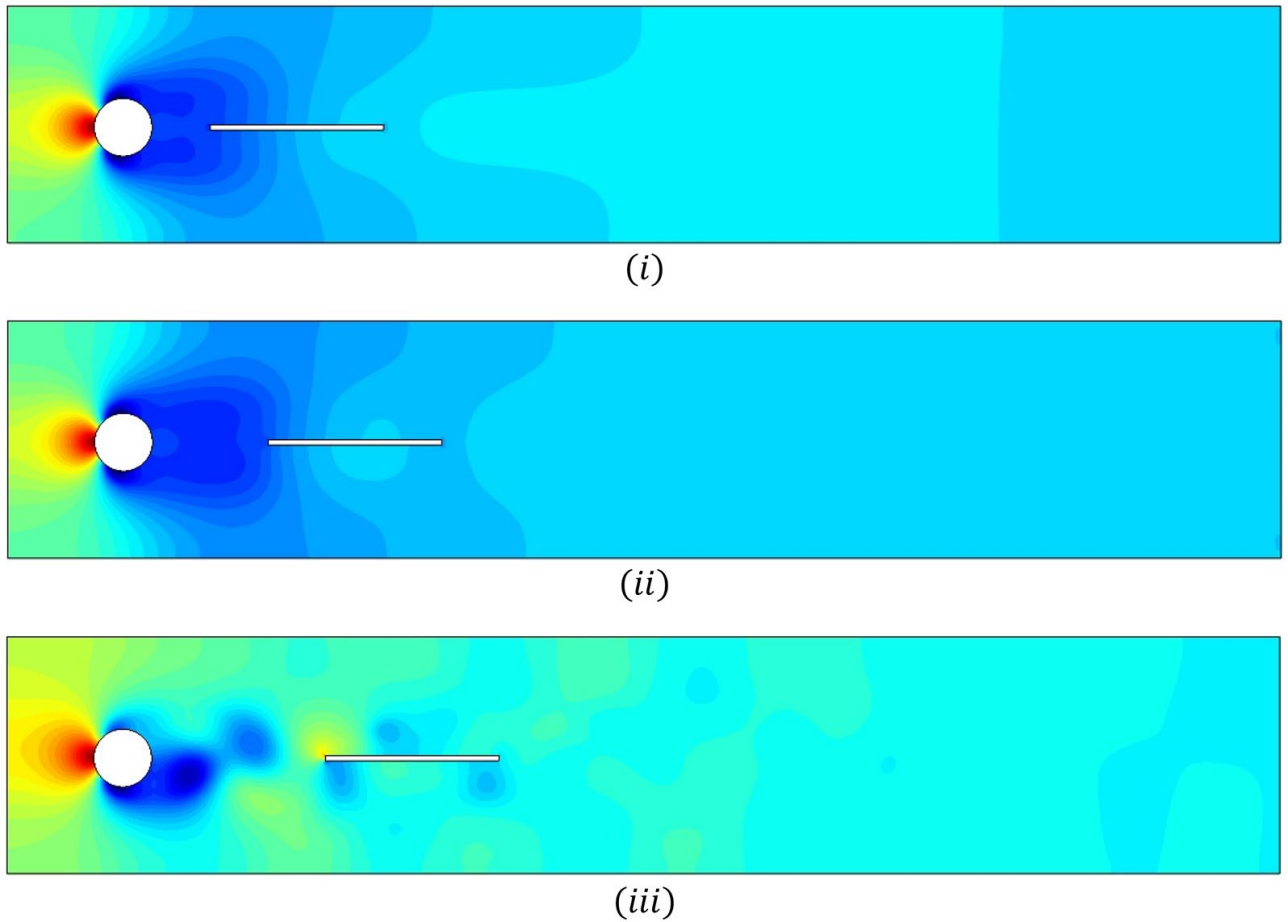
Influence of detached splitter plate (length $$L = 0.3)$$ on velocity, placed at gaps (**i**) $$G_{1} = 0.1,$$ (**ii**) $$G_{2} = 0.2,$$ (**iii**) $$G_{3} = 0.3$$ with $$Re = 100$$ at $$\overline{t} = 10.$$

The flow visualizations for fluid motion are shown in this section. Figure 7i–iii illustrates the total pressure for various splitter plate lengths and Fig. 8i–iii for splitter plate of fixed length $\tilde{L} = 0.3$.The pressure is maximum at the stagnation point and becomes minimum when fluid bifurcates around the circular obstacle. For the first case, each figure shows effects for splitter plate length varying from $0.1 - 0.3.$ The non-linearity of pressure is observed in Fig. 7i, as the plate of shortest length $L_{1} = 0.1$, affects periodicity negligibly. On the other hand, the plates of lengths $L_{2} = 0.2, and L_{3} = 0.3$ overcome periodicity so that pressure becomes linear efficiently, as shown in Fig. 7ii, iii. The second case, shown in Fig. 8i–iii, implies that the gap between the splitter plate and obstacle is adjusted in such a way that for a lesser gap $\left( {G_{1} = 0.1, G_{2} = 0.2} \right)$ between obstacle and plate, the nonlinear pressure profile becomes linear just after the fluid passes over the obstacle. For the maximum gap $G_{3} = 0.3$, the plate has lesser effects. It is noticed that the pressure between the gap is also minimum (Fig. 8i–iii).

**Figure 9:**
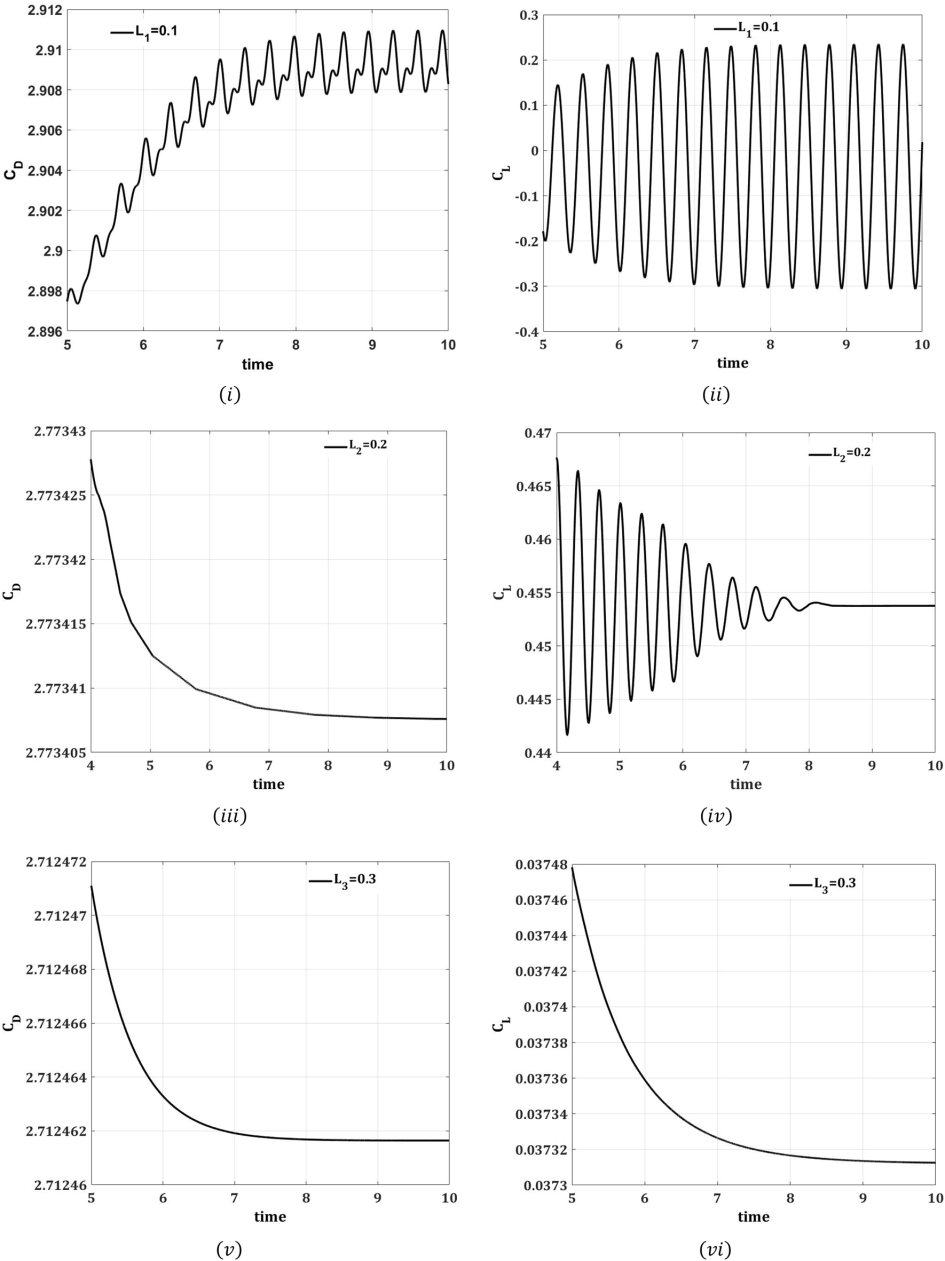
Impact of attached splitter plate on drag coefficient $(C_{{\text{D}}}$) and lift coefficient ($C_{{\text{L}}} )$ w.r.t time $\left( {\overline{t}} \right)$, step size $\Delta t = 0.001.$

**Figure 10:**
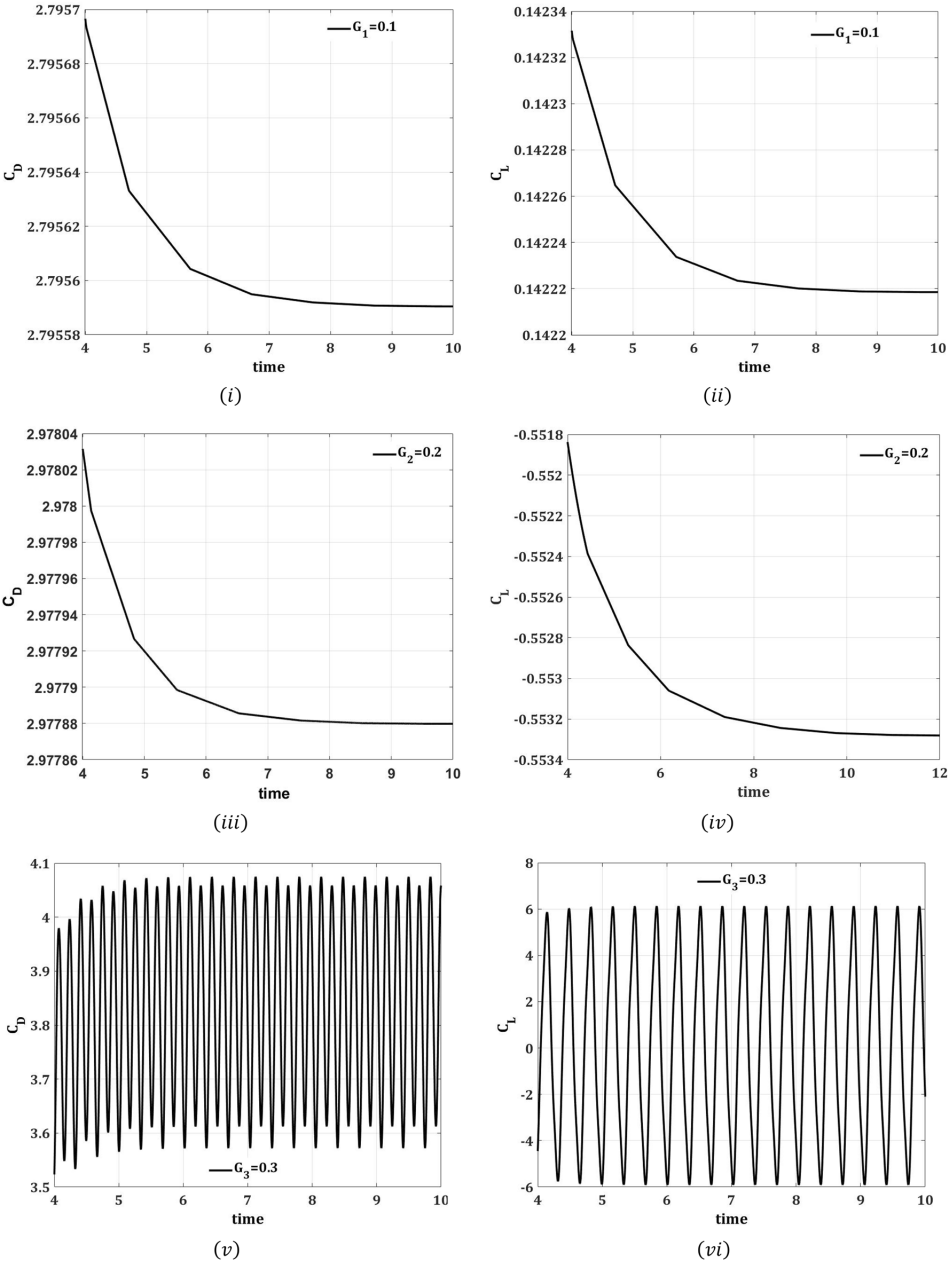
Impact of splitter plate with various gap separations $G_{i} , i = 0.1,0.2,0.3$ on drag coefficient $\left( {C_{{\text{D}}} } \right)$ and lift coefficient $\left( {C_{{\text{L}}} } \right)$, w.r.t time $\left( {\overline{t}} \right)$, step size $\Delta t = 0.001$.

The drag and lift forces are induced here in a channel flow because of the presence of an obstacle of circular shape centered at $\left( {0.2,0.2} \right)\;{\text{m}}$. These hydrodynamic quantities are produced because of the pressure and viscous forces imposed on the obstacle. In an unsteady flow regime, oscillation in both drag and lift forces has occurred which is a result of the vortex shedding formation in the flow. This research aims to control these hydrodynamic forces over a circular obstacle in an unsteady flow regime. Figures 9i–vi and 10i–vi show the time signals of the drag and lift coefficients for two different cases at $Re = 100.$ It is evident from Fig. 9i–vi that the drag coefficient is decreased by increasing the length of the plate, whereas the value of the lift coefficient is enhanced. It is well understood that for greater splitter plate length, there is a quick separation of flow and also away from the front of the circular obstacle. Figure 10i–vi depicts the impact of the second case on drag and lift coefficients. The splitter plate located at the lesser gap from the obstacle has a great impact on the reduction of drag coefficient and fluctuating values of lift coefficient. From the Fig. 9ii–vi, it is concluded that the presence of splitter plate has suppressed the vortex shedding and flow regime turns out to be steady as is evident from the non-oscillatory drag and lift coefficients.

## Conclusions

The fluid–solid interaction phenomena became so popular in the last decades due to its vast applications, especially in engineering fields. The damages caused by the unsteady forces (which occur because of this interaction) are very costly. For avoiding disastrous incidents, control devices (passive or active) are highly required. Throughout this work, the control effect is investigated by using a passive device i.e., a splitter plate. The splitter plate is used in the current study in two ways: $\left( I \right)$ a splitter plate of various lengths is attached to the circular obstacle, $\left( {II} \right)$ a spitter plate of fixed length at various gap separations from the obstacle. Using the Navier-Stokes equation in two-dimension and boundary constraints, the physical problem is mathematically expressed. A reliable computational tool, the finite element method is required for the current investigation. The results are shown using graphical patterns. The velocity and pressure variations are represented graphically. The reduction in drag coefficient and variation in lift coefficient are also represented. The important findings are as follows:

- A comparison of drag and lift coefficient has been done with and without a splitter plate.
- In presence of a splitter plate, a reduction in drag coefficient has been observed.
- For a smaller length of the plate, the lift is periodic; however, with an increase in the length, it converges to a fixed value and loses its periodic behavior.
- For smaller gap separation, a greater reduction in drag coefficient is observed.
- For larger gap separation, the lift is periodic as well as the drag coefficient.

## Data Availability

The datasets used and/or analysed during the current study available from the corresponding author on reason-able request.

## References

[1] Choi H, Jeon WP, Kim J (2008). Control of flow over a bluff body. Annu. Rev. Fluid Mech..

[2] Rashidi S, Hayatdavoodi M, Esfahani JA (2016). Vortex shedding suppression and wake control: A review. Ocean Eng..

[3] Zdravkovich MM (1981). Review and classification of various aerodynamic and hydrodynamic means for suppressing vortex shedding. J. Wind Eng. Ind. Aerodyn..

[4] Bearman PW (1965). Investigation of the flow behind a two-dimensional model with a blunt trailing edge and fitted with splitter plates. J. Fluid Mech..

[5] Kwon K, Choi H (1996). Control of laminar vortex shedding behind a circular cylinder using splitter plates. Phys. Fluids.

[6] Assi GRS, Bearman PW, Kitney N (2009). Low drag solutions for suppressing vortex-induced vibration of circular cylinders. J. Fluids Struct..

[7] Gu F, Wang JS, Qiao XQ, Huang Z (2012). Pressure distribution, fluctuating forces and vortex shedding behavior of circular cylinder with rotatable splitter plates. J. Fluids Struct..

[8] Bao Y, Tao J (2013). The passive control of wake flow behind a circular cylinder by parallel dual plates. J. Fluids Struct..

[9] Serson D, Meneghini JR, Carmo BS, Volpe EV, Gioria RS (2014). Wake transition in the flow around a circular cylinder with a splitter plate. J. Fluid Mech..

[10] Yu Y, Xie F, Yan H, Constantinides Y, Oakley O, Karniadakis GE (2015). Suppression of vortex-induced vibrations by fairings: A numerical study. J. Fluids Struct..

[11] Xie F, Yu Y, Constantinides, Triantafyllou MS, Karniadakis GE (2015). “U-shaped fairings suppress vortex-induced vibrations for cylinders in cross-flow. J. Fluid Mech..

[12] Baarholm R, Skaugset K, Lie H, Braaten H (2015). Experimental studies of hydrodynamic properties and screening of riser fairing concepts for deep water applications. Int. Conf. Offshore Mech. Arctic Eng..

[13] Dong S, Triantafyllou GS, Karniadakis GE (2008). Elimination of vortex streets in bluff-body flows. Phys. Rev. Lett..

[14] Chen WL, Xin DB, Xu F, Li H, Ou JP, Hu H (2013). Suppression of vortex-induced vibration of a circular cylinder using suction-based flow control. J. Fluids Struct..

[15] Korkischko I, Meneghini JR (2012). Suppression of vortex-induced vibration using moving surface boundary-layer control. J. Fluids Struct..

[16] Baek H, Karniadakis GE (2009). Suppressing vortex-induced vibrations via passive means. J. Fluids Struct..

[17] Pontaza JP, Menon RG (2008). Numerical simulations of flow past an aspirated fairing with three degree-of-freedom motion. Int. Conf. Offshore Mech. Arctic Eng..

[18] Corson D, Cosgrove S, Constantinides Y (2014). Application of CFD to predict the hydrodynamic performance of offshore fairing designs. Int. Conf. Offshore Mech. Arctic Eng..

[19] Allen, D. W., Henning, D. L., Haws, J. H., McMillan, D. W. & McDaniel, R. B. Partial helical strake for vortex-induced-vibrationsuppression, US Patent No. 6,561,734. Washington, DC: U.S. Patent and Trademark Office (2003).

[20] Trim AD, Braaten H, Lie H, Tognarelli MA (2005). Experimental investigation of vortex-induced vibration of long marine risers. J. Fluids Struct..

[21] Owen JC, Bearman PW, Szewczyk AA (2001). Passive control of VIV with drag reduction. J. Fluids Struct..

[22] Bearman P, Branković M (2004). Experimental studies of passive control of vortex-induced vibration. Eur. J. Mech. B/Fluids..

[23] Triantafyllou GS, Triantafyllou MS, Chryssostomidis C (1986). On the formation of vortex streets behind stationary cylinders. J. Fluid Mech..

[24] Hwang JY, Yang KS, Sun SH (1994). Reduction of flow-induced forces on a circular cylinder using a detached splitter plate. Phys. Fluids..

[25] Akilli H, Sahin B, Tumen NF (2005). Suppression of vortex shedding of circular cylinder in shallow water by a splitter plate. Flow Meas. Instrum..

[26] Ozono S (2003). Vortex suppression of the cylinder wake by deflectors. J. Wind Eng. Ind. Aerodyn..

[27] Serson D, Meneghini JR, Carmo BS, Volpe EV, Assi GRS (2015). Numerical study of the flow around a circular cylinder with dual parallel splitter plates. Instabil. Control Mass. Sep. Flows.

[28] Rockwell D, Unal MF (1987). On vortex formation from a cylinder. Part 2. Control by splitter-plate interference. J. Fluid Mech..

[29] Cete AR, Unal MF (1992). Effects of splitter plate on wake formation from a circular cylinder: A discrete vortex simulation. Comp. Fluid Dyn..

[30] Gao DL, Chen G-B, Huang YW, Chen WL, Li H (2020). Flow characteristics of a fixed circular cylinder with an upstream splitter plate: On the plate-length sensitivity. Exp. Therm. Fluid Sci..

[31] Chen WL, Chen GB, Xu F, Huang Y, Gao DL, Li H (2020). Suppression of vortex-induced vibration of a circular cylinder by a passive-jet flow control. J. Wind Eng. Ind. Aerodyn..

[32] Chen G, Chen W, Min X, Gao D (2022). A coupled model for vortex induced vibration of a circular cylinder with and without passive-jet flow control. J. Fluids Struct..

[33] Schäfer M, Turek S, Durst F, Krause E, Rannacher R (1996). Benchmark computations of laminar flow around a cylinder. NNFM.

[34] Mahmood R, Bilal S, Majeed AH, Khan I, Nisar KS (2020). Assessment of pseudo-plastic and dilatant materials flow in channel driven cavity: Application of metallurgical processes. J. Mater. Res. Technol..

[35] Mahmood R, Majeed AH, Ain QU, Awrejcewicz J, Khan I, Shahzad H (2022). Computational analysis of fluid forces on an obstacle in a channel driven cavity: Viscoplastic material based characteristics. Materials.

[36] Schenk, O., Gärtner, K. PARDISO. In *Encyclopedia of Parallel Computing* (ed. Padua, D.) (Springer, Boston, 2011).

